# Green Synthesized Montmorillonite/Carrageenan/Fe_3_O_4_ Nanocomposites for pH-Responsive Release of Protocatechuic Acid and Its Anticancer Activity

**DOI:** 10.3390/ijms21144851

**Published:** 2020-07-09

**Authors:** Yen Pin Yew, Kamyar Shameli, Shaza Eva Mohamad, Kar Xin Lee, Sin-Yeang Teow

**Affiliations:** 1 Chemical Energy Conversion and Application (ChECA), Malaysia-Japan International Institute of Technology (MJIIT), Universiti Teknologi Malaysia, Jalan Sultan Yahya Petra, Kuala Lumpur 54100, Malaysia; yewyenpin@gmail.com (Y.P.Y.); shaza@utm.my (S.E.M.); hanakarxinlee@gmail.com (K.X.L.); 2 Department of Medical Sciences, School of Healthcare and Medical Sciences, Sunway University, Jalan, Universiti, Bandar Sunway, Selangor Darul Ehsan 47500, Malaysia

**Keywords:** anticancer, carrageenan, iron oxide, Fe_3_O_4_ nanocomposites, montmorillonite, protocatechuic acid, drug delivery

## Abstract

Discovery of a novel anticancer drug delivery agent is important to replace conventional cancer therapies which are often accompanied by undesired side effects. This study demonstrated the synthesis of superparamagnetic magnetite nanocomposites (Fe_3_O_4_-NCs) using a green method. Montmorillonite (MMT) was used as matrix support, while Fe_3_O_4_ nanoparticles (NPs) and carrageenan (CR) were used as filler and stabilizer, respectively. The combination of these materials resulted in a novel nanocomposite (MMT/CR/Fe_3_O_4_-NCs). A series of characterization experiments was conducted. The purity of MMT/CR/Fe_3_O_4_-NCs was confirmed by X-ray diffraction (XRD) analysis. High resolution transmission electron microscopy (HRTEM) analysis revealed the uniform and spherical shape of Fe_3_O_4_ NPs with an average particle size of 9.3 ± 1.2 nm. Vibrating sample magnetometer (VSM) analysis showed an M_s_ value of 2.16 emu/g with negligible coercivity which confirmed the superparamagnetic properties. Protocatechuic acid (PCA) was loaded onto the MMT/CR/Fe_3_O_4_-NCs and a drug release study showed that 15% and 92% of PCA was released at pH 7.4 and 4.8, respectively. Cytotoxicity assays showed that both MMT/CR/Fe_3_O_4_-NCs and MMT/CR/Fe_3_O_4_-PCA effectively killed HCT116 which is a colorectal cancer cell line. Dose-dependent inhibition was seen and the killing was enhanced two-fold by the PCA-loaded NCs (IC_50_–0.734 mg/mL) compared to the unloaded NCs (IC_50_–1.5 mg/mL). This study highlights the potential use of MMT/CR/Fe_3_O_4_-NCs as a biologically active pH-responsive drug delivery agent. Further investigations are warranted to delineate the mechanism of cell entry and cancer cell killing as well as to improve the therapeutic potential of MMT/CR/Fe_3_O_4_-NCs.

## 1. Introduction

Cancer is a primary health problem and it is one of the leading causes of death globally [1]. According to the International Agency for Research on Cancer (IARC) GLOBOCAN 2018 report the cancer incidence and mortality statistics showed that there were 18.1 million new cancer cases and 9.6 million cancer-related deaths in 2018 [2]. Compared to 2012, which had 14.1 million newly diagnosed cancer cases and 8.2 million cancer-related death cases [3], it is apparent that the cancer incidence and mortality rates have experienced a drastic leap. Chemotherapy remains one of the common therapeutic modalities for cancer despite the fact it brings along side effects which may damage multiple body organs and aggravate the treatment [4]. Hence, the development of targeted drug delivery systems is crucial to solve this problem. Target-specific drug carrier systems are capable of delivering drugs to their target site as well as reducing the adverse side effects [5].

The use of nanotechnology in various biomedical applications has shown promise in the past few years. To date, there are more than 250 nanomedicine products and 50 of them are available on the market for clinical use [6]. The development of nanotechnology has advanced several biomedical applications such as disease diagnosis, drug delivery, imaging and sensing in living organisms [7,8]. Some of the examples include application of gold NPs in biosensors for diagnosis of foot and mouth disease virus [9], application of graphene oxide NCs for imaging breast cancer via intratumoral administration [10], and utilization of multifunctional mesoporous silica NPs for cancer-specific drug delivery [11]. Inorganic nanomaterials such as magnetite (Fe_3_O_4_) has been widely studied for its biomedical application such as magnetic resonance imaging (MRI) [12], magnetic hyperthermia [13] and drug delivery [14,15]. Some of the key advantages of using Fe_3_O_4_ is its high biocompatibility, biodegradability and non-toxicity [16,17,18]. Besides, Fe_3_O_4_ has superparamagnetic properties which can be beneficial in the use of targeted therapies as the magnetic Fe_3_O_4_ can be specifically directed to a target site using a magnetic field. However, some of the potential problems using the bare Fe_3_O_4_ NPs are high-level oxidation, poor bioavailability and possible agglomeration [19].

In this study, Fe_3_O_4_-NCs was synthesized via a green method. MMT was utilized as a matrix support due to its advantageous characteristics, such as high cation exchange capacity, good absorbance ability and drug-carrying capability [20] which may enhance the drug delivery efficiency. CR was used as stabilizing agent [21] to stabilize the Fe_3_O_4_ NPs in the NCs. CR is the major component found in most of the seaweeds and it is composed of a linear galactose backbone which is an anionic sulphated polysaccharide [22]. CR has high biocompatibility and consolidation behavior as well as various biological activities such as anticancer and immunomodulatory actions. CR can also act as an excipient in drug formulations such as in tablets for controlled release of drug, CR-drug composite for sustained release and CR-based gelling drug [23]. In the current study, we hypothesized that the combination of MMT and CR for Fe_3_O_4_-NCs synthesis (MMT/CR/Fe_3_O_4_-NCs) could enhance the drug delivery efficiency and the corresponding biological action. As a sample model, an anticancer drug, protocatechuic acid (PCA) was incorporated into the NCs (MMT/CR/Fe_3_O_4_-PCA) to investigate the drug loading efficiency and the anticancer activity. PCA is a simple phenolic compound and it is commonly found in edible plants. PCA exhibits various biological and pharmacological activities including anticancer action [24]. It has been previously shown to inhibit cell proliferation in several cancers including breast (MCF7), lung (A549), liver (HepG2), cervix (HeLa) and prostate (LNCap) cancers [25]. Herein, we evaluated the anticancer action of the MMT/CR/Fe_3_O_4_-PCA against the HCT116 colon cancer cell line.

## 2. Results

### 2.1. Synthesis of MMT/CR/Fe_3_O_4_-NCs

**Figure 1:**
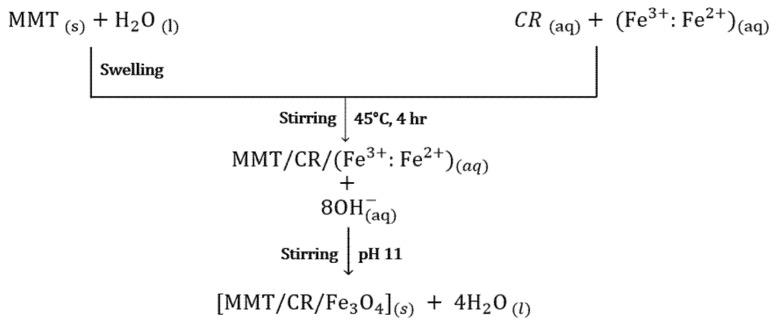
Chemical reactions of MMT, CR, Fe^2+^ and Fe^3+^ chloride salts solution in MMT/CR/Fe_3_O_4_-NCs synthesis.

The Fe^3+^/Fe^2+^ chloride salts and CR solution were added together into the MMT solution and stirred continuously to allow CR and Fe^3+^/Fe^2+^ ions solutions to mix into the interlayer space of MMT. To allow for this phenomenon to occur, MMT powder was first dissolved into deionized water [26]. After that, the freshly prepared 1 M NaOH was added with continuous stirring to synthesize MMT/CR/Fe_3_O_4_-NCs. The color of the solution turned dark brown after adding NaOH which indicated the formation of Fe_3_O_4_. Using an external magnet, the NPs in the solution could be separated which showed the magnetic properties of the synthesized MMT/CR/Fe_3_O_4_-NCs. The chemical reactions of MMT/CR/Fe_3_O_4_-NCs synthesis is presented in Figure 1.

### 2.2. X-Ray Diffraction (XRD) Analysis

**Figure 2:**
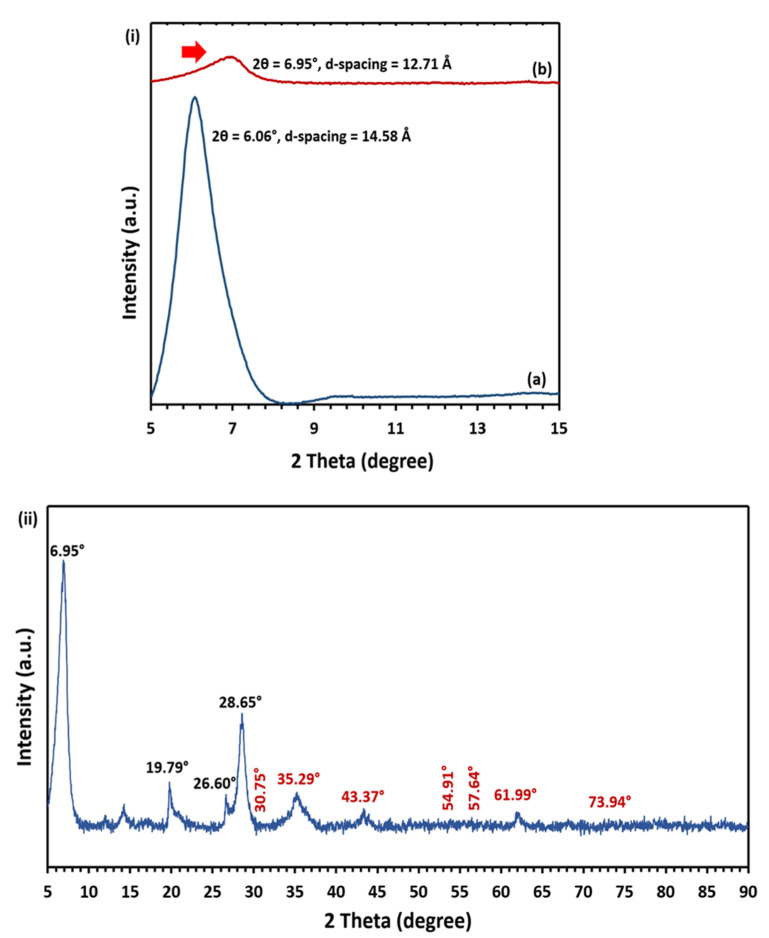
(**i**) XRD patterns of (a) pristine MMT and (b) MMT/CR/Fe_3_O_4_-NCs at 2θ = 5° to 15°, red arrow shows the right shift of the peak; (**ii**) full XRD pattern of MMT/CR/Fe_3_O_4_-NCs.

The XRD patterns of MMT/CR/Fe_3_O_4_-NCs are presented in Figure 2. Figure 2 (i) shows the major peak of MMT for (a) pristine MMT and (b) MMT/CR/Fe_3_O_4_-NCs at 2θ = 5° to 15°. The peak of MMT of MMT/CR/Fe_3_O_4_-NCs showed a strong (001) reflection at 2θ = 6.95° with d_(100)_-spacing of 12.71 Å. This showed the right shifting of peak (shown by red arrow in Figure 2i) and decrease in d-spacing as compared to pristine MMT which had 2θ at 6.06° with d_(001)_-spacing of 14.58 Å. The full XRD pattern of MMT/CR/Fe_3_O_4_-NCs is shown in Figure 2 (ii). The peaks near 2θ = 6.95°, 19.79° and 28.65° corresponded to the MMT. The peak at 2θ = 26.60° suggested the impurity most likely due to the quartz in the clay (JCDPS file no.: 00-005-0490). 

Besides, diffraction peaks were observed at 2θ = 30.75°, 35.29°, 43.37°, 54.91°, 57.64°, 61.99° and 73.94° respectively (shown by red text in Figure 2ii). These peaks were attributed to the crystal planes of (200), (311), (400), (422), (511), (440) and (533) respectively, which matched the standard XRD pattern of Fe_3_O_4_ (JCDPS file no.: 00-019-0629).

### 2.3. High-Resolution Transmission Electron Microscopy (HRTEM) Analysis

**Figure 3:**
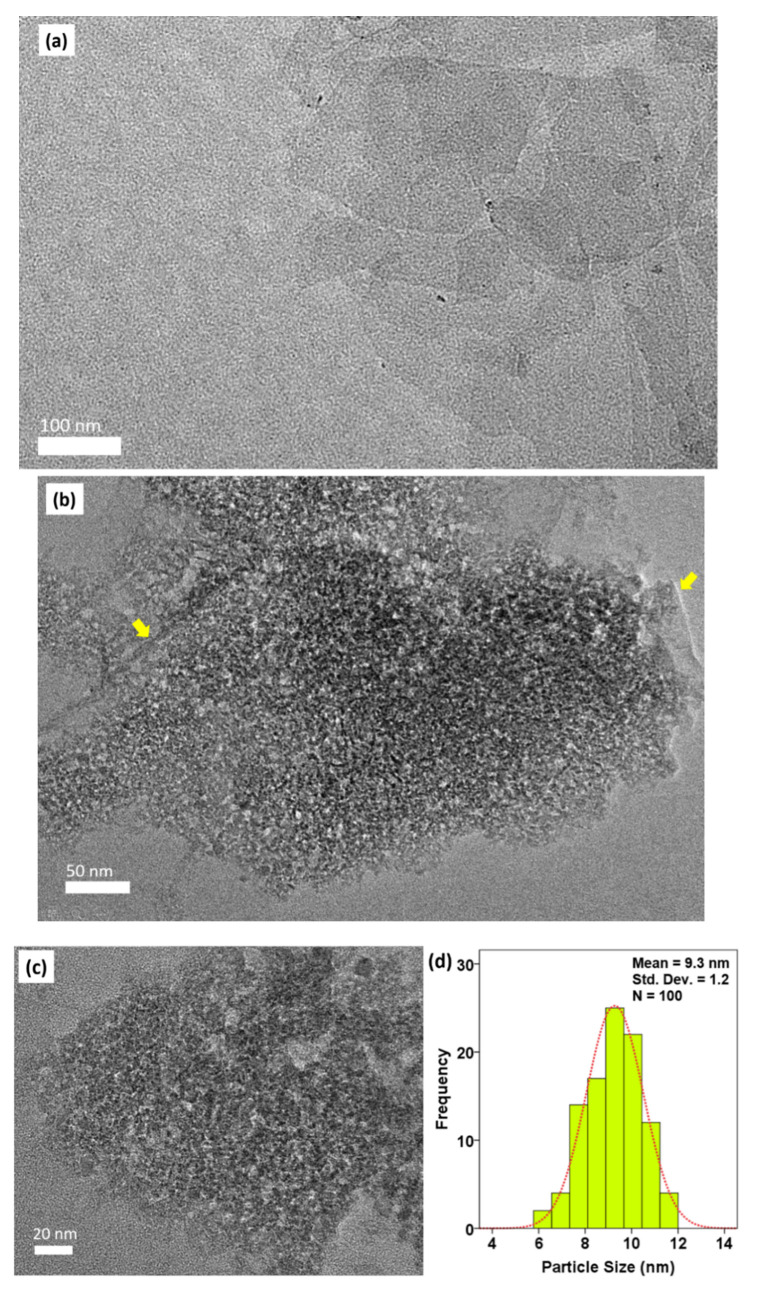
TEM images of (**a**) pristine MMT, scale bar represents 100 nm; (**b**) MMT/CR/Fe_3_O_4_-NCs at higher magnification, scale bar represents 50 nm, yellow arrows show the depositing of Fe_3_O_4_-NPs on the lamellar structure of MMT; and (**c**) MMT/CR/Fe_3_O_4_-NCs at lower magnification, scale bar represents 20 nm; (**d**) Size distribution histogram of MMT/CR/Fe_3_O_4_-NCs.

The particle size and shape of MMT/CR/Fe_3_O_4_-NCs were determined by TEM analysis. TEM image of pristine MMT is shown in Figure 3a which demonstrated a sheet-like structure with multi layered platelets, which is the nature of scattering and distribution of layered silicates [27].

Figure 3b,c show the TEM images of MMT/CR/Fe_3_O_4_-NCs at higher and lower magnification, respectively. In Figure 3b, Fe_3_O_4_-NPs were deposited on the lamellar structure of MMT as shown by yellow arrows. A size distribution histogram was plotted and the average particle size was 9.3 ± 1.2 nm from the measurement of 100 NPs (Figure 3d). The NPs appeared to have a uniform spherical shape with a small size distribution.

### 2.4. Field Emission Scanning Electron Microscopy with Energy Dispersive X-Ray Spectroscopy (FESEM-EDX) Analysis

**Figure 4:**
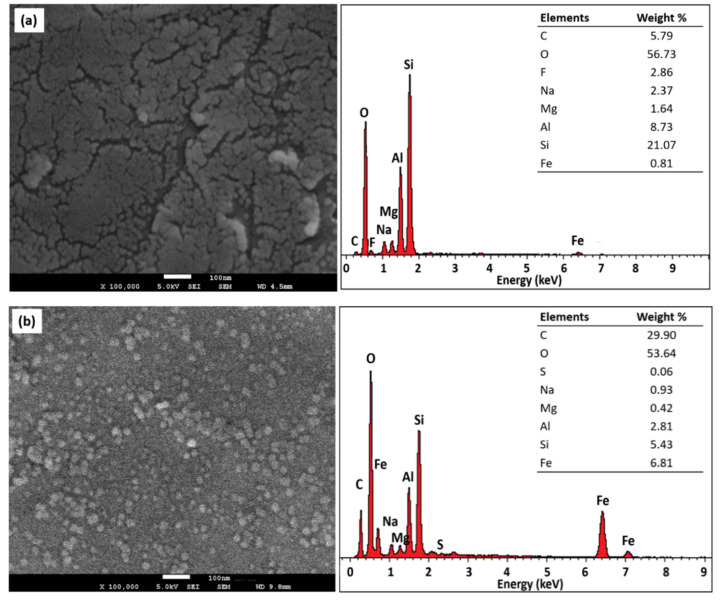
FESEM images and EDX spectra of (**a**) pristine MMT and (**b**) MMT/CR/Fe_3_O_4_-NCs (inset: elements composition).

FESEM analysis was performed to study the surface morphology of samples and the FESEM images were viewed and presented at the magnification of X100,000. The FESEM images of pristine MMT and MMT/CR/Fe_3_O_4_-NCs with their respective EDX spectra are shown in Figure 4a,b, respectively. Spherical particles were observed on the surface of MMT and MMT/CR/Fe_3_O_4_-NCs, in which the particles were the tactoids of MMT. 

The EDX spectrum of MMT/CR/Fe_3_O_4_-NCs showed three primary peaks at around 0.6, 6.3 and 7.0 keV as shown in Figure 4b, which attributed to the binding energies of the iron [28]. Thus, the formation of Fe_3_O_4_ was confirmed. In addition, sulfur (S) was found in MMT/CR/Fe_3_O_4_-NCs at low weight percentage (0.06%), while carbon (C) was found in a high weight percentage (29.90%). 

These results confirmed the presence of CR in the NCs. On top of that, the decreased weight percentage of sodium (Na), magnesium (Mg), aluminium (Al) and silicon (Si) in MMT/CR/Fe_3_O_4_-NCs was observed when compared to those in pristine MMT. This might be due to the ion exchange and interaction of MMT, CR and Fe_3_O_4_-NPs.

### 2.5. Vibrating Sample Magnetometer (VSM) Analysis

**Figure 5:**
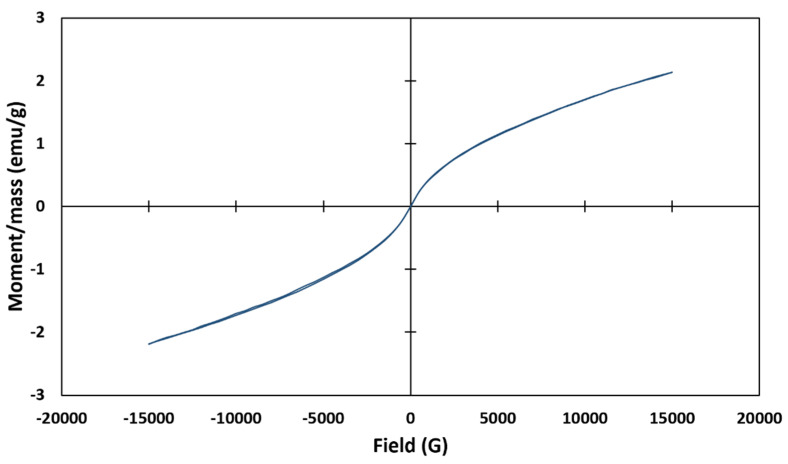
Magnetization curve of MMT/CR/Fe_3_O_4_-NCs.

To confirm the magnetic properties of MMT/CR/Fe_3_O_4_-NCs, VSM analysis was carried out and the magnetization curve is presented in Figure 5. The hysteresis loop of MMT/CR/Fe_3_O_4_-NCs showed the saturation magnetization of (M_s_) value of 2.16 emu/g with negligible coercivity (H_c_) 1.42 Oe. This confirmed the superparamagnetic characteristic of MMT/CR/Fe_3_O_4_-NCs.

### 2.6. Drug Loading and Release

**Figure 6:**
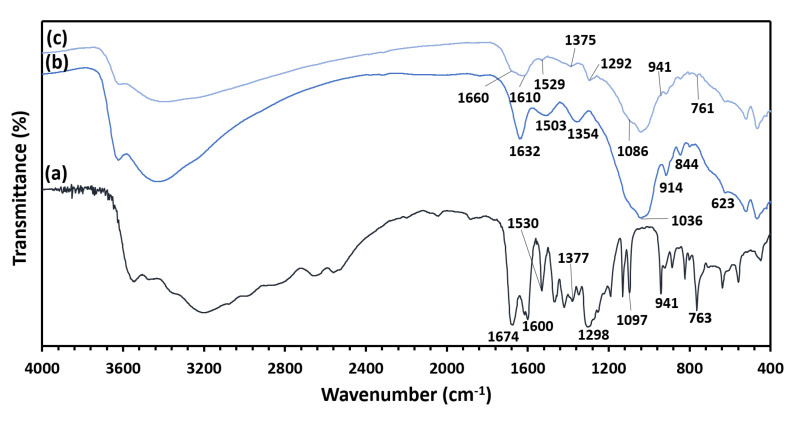
FTIR spectra of (**a**) PCA, (**b**) MMT/CR/Fe_3_O_4_-NCs and (**c**) MMT/CR/Fe_3_O_4_-PCA.

Drug loading was assessed by FTIR analysis. The FTIR spectra of PCA, MMT/CR/Fe_3_O_4_-NCs and MMT/CR/Fe_3_O_4_-PCA are presented in Figure 6. The absorption peaks of PCA at 1674 cm^−1^ corresponded to carboxylic acid C=O group, 1600, 1530 and 1377 cm^−1^ are aromatic C-C stretching bands, 1298 and 1097 cm^−1^ are C-O stretching bands, 941 cm^−1^ is the OH bending vibration of the carboxylic acid group and 763 cm^−1^ is the C-H bending band, respectively. These peaks were also found in MMT/CR/Fe_3_O_4_-PCA shifted to 1660, 1610, 1529, 1375, 1292, 1086, 941 and 761 cm^−1^ at low intensity [29]. In addition, the peaks of MMT/CR/Fe_3_O_4_-NCs at 1632, 1503 and 1354 cm^−1^ correspond to the O-H stretching band, -C=O stretching band, and sulphated polysaccharide, respectively, which are not found in MMT/CR/Fe_3_O_4_-PCA. Lastly, the intensity of peaks at 1036 (Si-O stretching), 914, 844, 761 (Al-O, Fe-O and Mg-O stretching) and 623 cm^−1^ (Al-OH) [30] decreased in MMT/CR/Fe_3_O_4_-PCA. 

**Figure 7:**
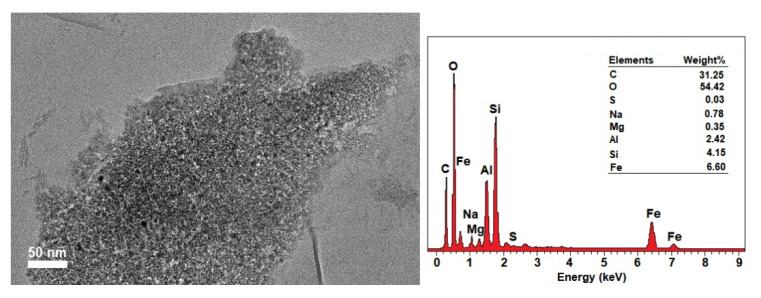
TEM image (scale bar represents 50 nm) and EDX spectrum of MMT/CR/Fe_3_O_4_-PCA (inset: elements composition).

After the successful loading, MMT/CR/Fe_3_O_4_-PCA was examined by TEM/EDX analysis (Figure 7). The structure of NCs was not affected and the lamellar structure of MMT still remained similar to that observed in MMT/CR/Fe_3_O_4_-NCs (Figure 3b). The EDX spectrum of MMT/CR/Fe_3_O_4_-PCA also showed similar finding with those in MMT/CR/Fe_3_O_4_-NCs (Figure 4b), but in different weight percentages. After the drug loading, the weight percentage of C and O increased to 31.25% and 54.42%, respectively due to the PCA which comprises of oxygen (O), carbon (C) and hydrogen (H) [31].

**Figure 8:**
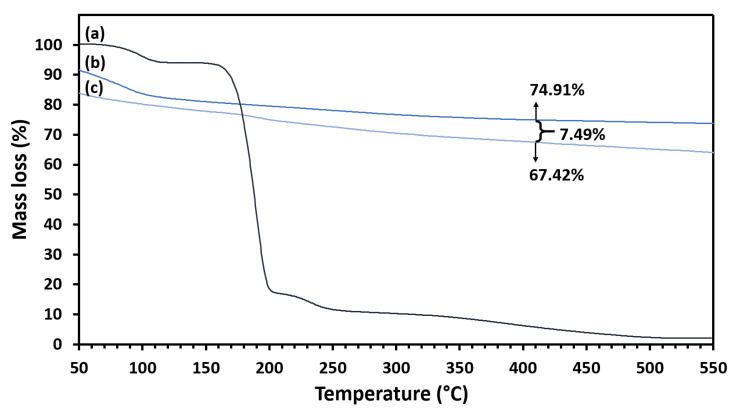
Thermograms of (**a**) PCA, (**b**) MMT/CR/Fe_3_O_4_-NCs and (**c**) MMT/CR/Fe_3_O_4_-PCA.

TGA was performed by comparing the mass loss occurred in PCA and MMT/CR/Fe_3_O_4_-PCA. Thermograms of PCA, MMT/CR/Fe_3_O_4_-NCs and MMT/CR/Fe_3_O_4_-PCA are presented in Figure 8. There are two thermal phenomena as shown in the thermogram of PCA. The first step was in the region of 50–142 °C while the second step was in the region of 210–282 °C. The mass loss of MMT/CR/Fe_3_O_4_-NCs and MMT/CR/Fe_3_O_4_-PCA was investigated at 410 °C as marked in Figure 8. 

**Figure 9:**
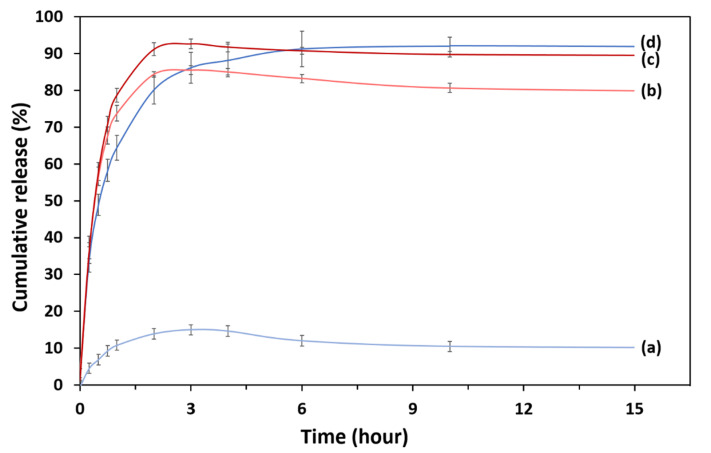
Drug release profile of (**a**) MMT/CR/Fe_3_O_4_-PCA at pH 7.4, (**b**) PCA at pH 7.4, (**c**) PCA at pH 4.8 and (**d**) MMT/CR/Fe_3_O_4_-PCA at pH 4.8.

Drug release studies were carried out in PBS adjusted to two different pH values: pH 4.8 and 7.4. The drug release profiles are presented in Figure 9. It was observed that PCA alone had a burst release of 85% and 92% at pH 7.4 and pH 4.8, respectively within the first 3 h. At pH 7.4, MMT/CR/Fe_3_O_4_-PCA showed burst release in the first 3 h with a lower percentage-15%. After 4 h, the drug release profile showed decreasing slope (Figure 9a) which could be due to the oxidation of PCA at pH 7.4. In contrast, MMT/CR/Fe_3_O_4_-PCA showed a more stable release profile at pH 4.8. Figure 9d shows that MMT/CR/Fe_3_O_4_-PCA took around 10 h to reach to a stable release profile which had a PCA release of 92%. 

### 2.7. Cytotoxicity Study

The cytotoxicity of samples was evaluated on colorectal cancer (HCT116) and colon normal (CCD112) cell lines. The IC_50_ (inhibitory concentration that kills 50% of cells) value is shown in Table 1. 

**Figure 10:**
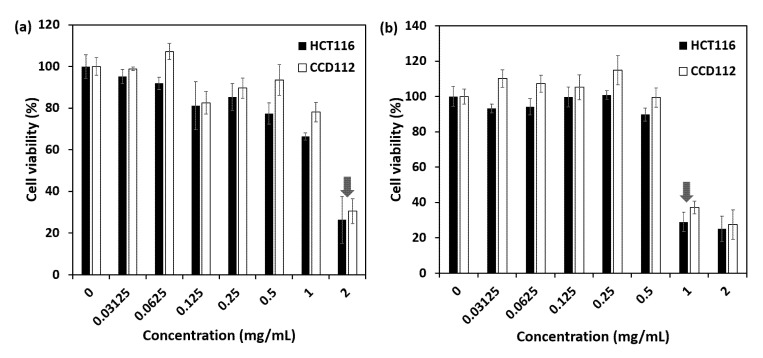
Cell viability of HCT116 and CCD112 cell lines after treatment of (**a**) MMT/CR/Fe_3_O_4_-NCs and (**b**) MMT/CR/Fe_3_O_4_-PCA at various concentrations. Red arrows show approximately 70% of growth inhibition of the tested cells.

Figure 10 shows the cytotoxicity of MMT/CR/Fe_3_O_4_-NCs and MMT/CR/Fe_3_O_4_-PCA. PCA was used as the control compound. In both tested cells lines, MMT/CR/Fe_3_O_4_-PCA exhibited approximately two-fold higher killing effect than the unloaded MMT/CR/Fe_3_O_4_-NCs based on the IC_50_ difference (Table 1). Both MMT/CR/Fe_3_O_4_-NCs and MMT/CR/Fe_3_O_4_-PCA had slightly higher killing activities against HCT116 than CCD112 cell lines (IC_50_ on HCT116 cells was lower than that in CCD112). MMT/CR/Fe_3_O_4_-NCs killed about 70% of HCT116 and CCD112 cells when treated at 2 mg/mL (shown by red arrow in Figure 10a). Similar killing efficiency was seen when the cells were treated with 1 mg/mL MMT/CR/Fe_3_O_4_-PCA (shown by red arrow in Figure 10b).

## 3. Discussion

XRD analysis was performed to study the crystallinity and phase purity of MMT/CR/Fe_3_O_4_-NCs. The right shifting of MMT peak was observed, where 2θ = 6.06° was shifted to 2θ = 6.95° after the modification. This phenomenon also implied the decrease in d-spacing of the interlayer of MMT. The shifting of MMT peak to a higher angle could be due to the crystallization of Fe_3_O_4_-NPs deposited outside the layer during intercalation, which suggests a breakdown of platelet that acquires agglomeration or leads to partial exfoliation [32]. Besides, the intensity of MMT peak of MMT/CR/Fe_3_O_4_-NCs was reduced compared to pristine MMT. This might be due to the interrupted MMT flakes in the process of NCs fabrication, thus the adjacent flakes were connected permanently [33]. Furthermore, the presence of Fe_3_O_4_ was confirmed without any impurity, such as hematite or maghemite, in which the diffraction peaks located at 2θ = 30.75°, 35.29°, 43.37°, 54.91°, 57.64°, 61.99°, 73.94°, corresponding to the (200), (311), (400), (422), (511), (440) and (533) planes, respectively, for the face-centered cubic (fcc) Fe_3_O_4_ lattice. This finding is in an agreement with the previous studies [34,35,36] which fitted well with the standard XRD pattern of Fe_3_O_4_. The shifting of peaks and the decreased intensity in XRD patterns indicated that there were strong interactions of MMT supports, CR and Fe_3_O_4_-NPs.

TEM results showed that the Fe_3_O_4_-NPs were deposited onto the lamellar structure of MMT. This finding is consistent with a previous study which showed the uniformly spherical shape of Fe_3_O_4_-NPs with the retained lamellar structure [37]. However, the image of lattice of NPs on MMT layer could not be examined as the MMT layers covered the surface of Fe_3_O_4_-NPs. 

FESEM images showed the spherical NPs on the surface of MMT and MMT/CR/Fe_3_O_4_-NCs, in which the NPs corresponded to the tactoids of MMT. The tactoids of pristine MMT formed agglomeration without any modification, while the tactoids of MMT/CR/Fe_3_O_4_-NCs showed lesser and separated agglomeration (Figure 4b). This result is consistent with a wood-plastic composites study, in which the modification process reduced the size of nanoclay tactoids and resulted in better NP dispersion [38]. The decreased size of tactoids might be related to the ion exchange of Na^+^ and Fe^2+^/Fe^3+^ ions from the MMT lamellar structure [39]. Besides, the binding energies of the iron was detected at about 0.6, 6.3 and 7.0 keV [28] as shown by the EDX spectra. The presence of Fe was verified by the increased weight percentage of Fe, from 0.81% (pristine MMT) to 6.81% (MMT/CR/Fe_3_O_4_-NCs). In contrast, the decreased weight percentage of Na (0.93%), Mg (0.42%), Al (2.81%) and Si (5.43%) in MMT/CR/Fe_3_O_4_-NCs was observed as compared to pristine MMT with weight percentage of Na (2.37%), Mg (1.64%), Al (8.73%) and Si (21.07%). This might be due to the ion exchange and interactions of MMT, CR and Fe_3_O_4_-NPs.

VSM results revealed the hysteresis loop of MMT/CR/Fe_3_O_4_-NCs with a M_s_ value of 2.16 emu/g. The low value might be due to the MMT layers which covered the surface of Fe_3_O_4_-NPs, as pointed out in TEM analysis. The presence of sulphur in the FESEM-EDX result could also confirm the masking of CR on Fe_3_O_4_-NPs. Low coercivity of MMT/CR/Fe_3_O_4_-NCs (1.42 Oe) was obtained which confirmed the superparamagnetic properties of MMT/CR/Fe_3_O_4_-NCs [40,41].

FTIR analysis was performed on PCA, MMT/CR/Fe_3_O_4_-NCs and MMT/CR/Fe_3_O_4_-PCA to study the functional groups present in each sample. From the spectra, the absorption peaks shifted and the peak intensity decreased after the drug loading. This could be due to the interaction between PCA and MMT/CR/Fe_3_O_4_-NCs during the drug loading processes [42,43]. This, in turn confirmed the successful loading of PCA onto MMT/CR/Fe_3_O_4_-NCs. In addition, TEM/EDX analysis was performed on MMT/CR/Fe_3_O_4_-PCA to determine the element compositions after drug loading. The weight percentage of C and O increased after the drug loading because PCA comprises of O, C and H. Thus, this finding further verified the successful PCA loading of MMT/CR/Fe_3_O_4_-NCs.

After confirmation of the PCA loading by FTIR, the amount of PCA loaded on MMT/CR/Fe_3_O_4_-NCs was measured before studying the drug release. In the region of 50–142 °C, decomposition of absorbed water occurred. In the second step of mass loss, there was a steep slope in the region of 210–282 °C, which could be attributed to the PCA decomposition [44]. Since the boiling point of PCA is 410 °C [31], the mass loss of MMT/CR/Fe_3_O_4_-NCs and MMT/CR/Fe_3_O_4_-PCA were determined at 410 °C to confirm the complete loss of PCA. The difference in percentage mass loss of these two samples indicated that there was 7.49% of PCA in MMT/CR/Fe_3_O_4_-PCA, indicating that the loading capacity was 7.49%. On the other hand, the calculated encapsulation efficiency was 26.5%, which is comparatively low. In fact, high drug loading efficiency is often difficult to obtain as drug loading is determined by the structure and physicochemical properties of the carrier material itself. Besides, drug loading process using physical and electrostatic adsorption could also result in low drug loading efficiency [45].

Based on the drug release profile, burst release was observed in the first 3 h for both PCA and MMT/CR/Fe_3_O_4_-PCA at pH 7.4. As the profile showed a decreasing slope after 4 h, it suggested that PCA is not stable at pH 7.4, which is consistent with a previous study [37]. At pH 4.8 mimicking the intracellular conditions of cancer cells, PCA and MMT/CR/Fe_3_O_4_-PCA released more PCA [46]. Comparatively, MMT/CR/Fe_3_O_4_-PCA revealed a more stable release profile, which took about 10h to reach to a stable release with 92% of release. From our finding, MMT/CR/Fe_3_O_4_-PCA is pH-dependent and more stable under acidic conditions [47]. Furthermore, the PCA release is stable, indicating the controlled and sustained release of PCA in the drug delivery system [48]. Therefore, MMT/CR/Fe_3_O_4_-NCs could be used as an effective pH- responsive drug delivery system for cancer therapies.

IC_50_ results showed that MMT/CR/Fe_3_O_4_-PCA resulted in a better inhibitory effect against HCT116 cells, which was approximately two-fold higher than MMT/CR/Fe_3_O_4_-NCs. These results highlighted the impact of loading PCA into MMT/CR/Fe_3_O_4_-NCs in killing the cancerous cells. Besides, MMT/CR/Fe_3_O_4_-NCs alone which possessed the potent anticancer activity could serve as a ‘double-edged sword’ when used as a nanocarrier for delivering drugs to cancerous cells. However, when compared to PCA alone, the cancer-killing action was reduced (Table 1). This can be partly explained by the low drug loading percentage, which was only 7.49%. As the selectivity (comparison of active dose against cancer and normal cells) of MMT/CR/Fe_3_O_4_-PCA was less than two-fold (Table 1), further modifications, particularly in nanocomposite-drug incorporation, are required to improve its use for cancer treatment. Since the MMT/CR/Fe_3_O_4_-NCs alone also possessed cytotoxic activity against normal cells (Table 1), the synthesis method must be further investigated and improved. For examples, the effect of different size, shape, configuration, surface charge and the type of coating of Fe_3_O_4_ nanocarriers on the toxicity of normal cells can be evaluated as previous studies have suggested that these properties play key roles in determining the nanocarrier’s toxicity profile [49,50,51]. In addition, strategies must be also looked into to improve the drug loading efficiency of the nanocomposites such as utilization of ultrasonic homogenizer for NCs and drug dispersion before shaking in water bath [52], and modification of MMT via ball-milling process to increase the specific surface areas of MMT and decrease its particle size [53].

## 4. Materials and Methods

### 4.1. Materials

Iron (III) chloride hexahydrate (FeCl_3_·6H_2_O, 97%), iron(II) chloride tetrahydrate (FeCl_2_·4H_2_O ≥ 99%) and protocatechuic acid (PCA) (≥97.0%) were obtained from Sigma Aldrich (St. Louis, MO, USA). Montmorillonite (MMT) was purchased from Kunipia (Tokyo, Japan). Carrageenan powder, sodium hydroxide (NaOH) and acetic acid were obtained from R&M Chemicals (London, UK). All materials were used without additional purification.

### 4.2. Cell culture

HCT116 (ATCC CCL-247) colorectal carcinoma and CCD112 (ATCC CRL-1541) colon normal cell lines were obtained from the American Type Culture Collection (ATCC, Manassas, VA, USA). Both cell lines were maintained in Dulbecco’s Modified Eagle’s medium (DMEM) supplemented with 10% fetal bovine serum (FBS) and 1% penicillin-streptomycin (Gibco, Carlsbad, CA, USA). Cellular cytotoxicity effect was determined by CellTiter 96 Aqueous One Solution or MTS reagent (#G3582, Promega, Madison, WI, USA) following the manufacturer’s instruction.

### 4.3. Preparation of MMT/CR/Fe_3_O_4_-NCs

MMT/CR/Fe_3_O_4_-NCs were synthesized using 1 g MMT powder, 0.1 g CR and 0.5 g Fe_3_O_4_. Firstly, 1 g of MMT powder was dissolved into deionized water with vigorous stirring for 2h at room temperature. Meanwhile, 0.1 g of CR powder was dissolved to 10 mL 1% acetic acid solution. Fe^3+^/Fe^2+^ solution at molar ratio of 2:1 was prepared and mixed with the CR solution. The mixture was then added into the MMT solution with continuous stirring for 4 h at 45 °C. The pH of solution was adjusted to pH 11 by adding the freshly prepared 1 M NaOH. The synthesized NCs were centrifuged and washed for several times using deionized water. Sample drying was performed in oven at 45 °C. The dried sample was kept in air-tight container for further characterizations. The experiments were conducted at ambient temperature.

### 4.4. Characterization

An XPert PRO X-ray diffractometer (XRD, PANalytical, Malvern, UK) was employed to determine the phase purity of the synthesized MMT/CR/Fe_3_O_4_-NCs at 2θ angle configuration scanning from 5° to 90° with scanning rate of 2θ/min. The surface morphology of MMT/CR/Fe_3_O_4_-NCs was studied using field-emission scanning electron microscopy (FESEM)-Energy Dispersive X-ray Spectroscopy (EDX) (JSM 7600F FESEM, JEOL, Tokyo, Japan) to identify the elemental composition. Particle size and morphology of MMT/CR/Fe_3_O_4_-NCs were examined using high resolution transmission electron microscopy (HRTEM), in which the samples were viewed under a JEOL JEM-2100F (Tokyo, Japan) using 400 mesh copper grids. The size of NPs was measured using ImageJ software [54,55]. The measurement was repeated for three times to ensure consistency. Magnetic properties of MMT/CR/Fe_3_O_4_-NCs was determined using a FCM-10 Vibrating Sample Magnetometer (VSM, Microsense, MA, USA) and the magnetization curve was recorded at room temperature. Fourier transform infrared (FT-IR) spectroscopy (IRTracer-100 FTIR spectrophotometer, Shimadzu, Kyoto, Japan) was performed to study the functional groups of biomolecules of samples using potassium bromide (KBr) method in the wavelength of 400–4000 cm^−1^. Thermogravimetric analysis (TGA) was utilized to identify the amount of drug loaded on samples, where the changes of weight loss (%) on drug loaded and unloaded samples were studied using a STA 449 F3 system (Netzsch, Selb, Germany; heating rate = 10 °C/min from 50 to 800 °C under nitrogen flow rate of 50 mL/min).

### 4.5. Drug Loading 

Protocatechuic acid (PCA) loading was performed using an encapsulation technique. Firstly, 0.1 g of MMT/CR/Fe_3_O_4_-NCs sample powder was dispersed in 25 mL phosphate buffer saline (PBS) solution. Next 25 mL of 6.2 mg/mL PCA solution was then added into the solution. The mixed solution was then incubated in a shaking water bath at 37 °C for 24 h. PCA-loaded MMT/CR/Fe_3_O_4_-NCs (named MMT/CR/Fe_3_O_4_-PCA) were collected after centrifugation and washed with PBS for three times. MMT/CR/Fe_3_O_4_-PCA were then dried and kept for drug release and cytotoxicity study. The encapsulation efficiency and loading capacity were calculated using equations as shown below [56,57]:

(1) $$\mathrm{Encapsulation}\;\mathrm{efficiency}\;\left(\%\right)=\frac{\mathrm{Total}\;\mathrm{drug}-\mathrm{Free}\;\mathrm{drug}}{\mathrm{Total}\;\mathrm{drug}}\times 100\;\left(\%\right)$$

(2) $$\mathrm{Loading}\;\mathrm{capacity}\;\left(\%\right)=\frac{\mathrm{Weight}\;\mathrm{of}\;\mathrm{drug}\;\mathrm{in}\;\mathrm{NCs}}{\mathrm{Weight}\;\mathrm{of}\;\mathrm{total}\;\mathrm{amount}\;\mathrm{of}\;\mathrm{NCs}}\times 100\;\left(\%\right)$$

Thermogravimetric analysis (TGA) was performed to determine the amount of PCA loaded onto MMT/CR/Fe_3_O_4_-NCs.

### 4.6. Drug Release Study

The in vitro dialysis bag technique was used to study the PCA release behavior from MMT/CR/Fe_3_O_4_-PCA as previously described [37,58,59]. This study was performed using PBS solution with an adjusted pH of 4.8 (intracellular lysosomal pH) [60] and 7.4 (human blood pH). Firstly, 25 mg of sample was dispersed in 5 mL of PBS solution in pH 4.8 or 7.4 and packed into a dialysis bag. The dialysis bag was then placed in 50 mL PBS (pH 4.8 or 7.4) with 100 rpm continuous stirring at 37 °C. One mL of released medium was collected at different time intervals (0, 0.25, 0.5, 0.75, 1, 2, 3, 4, 6, 10, 24 h) and immediately replenished by 1 mL fresh PBS in either pH 4.8 or 7.4. For the control study, 25 mg of free PCA was utilized. Each sample was repeated three times for both pH conditions. The amount of released PCA was determined by UV-Vis spectrophotometry (Shimadzu, Kyoto, Japan) at 288 nm. The cumulative drug release was calculated using equation as shown below:

(3) $$\mathrm{Drug}\;\mathrm{release}\;\left(\%\right)=\frac{\mathrm{Amount}\;\mathrm{of}\;\mathrm{drug}\;\mathrm{released}}{\mathrm{Amount}\;\mathrm{of}\;\mathrm{drug}\;\mathrm{loaded}}\times 100\;\left(\%\right)$$

### 4.7. Cytotoxicity Assay 

MTS assay was performed as previously described [59,61,62,63] to evaluate the cellular killing effect of NCs. For both HCT116 and CCD112, 5,000 cells per well (100 µL/well) were seeded onto 96-well plates and incubated overnight in a 5% CO_2_ incubator at 37 °C for complete adherence. Next day, 2-fold serially diluted NCs (100 µL/well) were added into the wells to make up the final concentration to 0, 0.03, 0.06, 0.125, 0.25, 0.5, 1 and 2 mg/mL. The plate was incubated for 72 h at 37 °C in the 5% CO_2_ incubator. Twenty µL of MTS reagent per well was then added into the plate and incubated for additional 3 h at 37 °C in the 5% CO_2_ incubator. The optical density (OD) was measured at 490 nm using a multimode microplate reader (Tecan, Mannedorf, Switzerland). The dose response graph was plotted by calculating the percent cell viability using equation below:

(4) $$\%\;\mathrm{Cell}\;\mathrm{viability}=\frac{\mathrm{OD}\;\mathrm{of}\;\mathrm{sample}\;\mathrm{well}\;\left(\mathrm{mean}\right)}{\mathrm{OD}\;\mathrm{of}\;\mathrm{control}\;\mathrm{well}\;\left(\mathrm{mean}\right)}\times \;100$$

In addition, inhibitory concentration causing 50% growth inhibition (IC_50_) was determined using an online calculator (https://www.aatbio.com/tools/ic50-calculator) as previously described [59,61,62,63].

## 5. Conclusions

MMT/CR/Fe_3_O_4_-NCs were successfully synthesized via a green approach in this study. The phase purity of MMT/CR/Fe_3_O_4_-NCs was confirmed by XRD analysis. TEM results revealed the lamellar structure of MMT and the spherical shape of the Fe_3_O_4_ NPs with an average particle size of 9.3 ± 1.2 nm. The superparamagnetic properties of MMT/CR/Fe_3_O_4_-NCs was confirmed by VSM, in which the M_s_ value was 2.16 emu/g with negligible coercivity. In a drug release study, MMT/CR/Fe_3_O_4_-PCA showed a more controlled drug release profile at pH 4.8 (92%) than pH 7.4 (15%). The anticancer assay showed that MMT/CR/Fe_3_O_4_-PCA had higher killing activity than MMT/CR/Fe_3_O_4_-NCs in HCT116 cells, which further confirmed the successful loading of PCA. The outcome of this study highly supports the potential of MMT/CR/Fe_3_O_4_-NCs to be used as drug delivery agents. In-depth modifications on the NCs are however required in future studies to further improve their anticancer activities and selectivity.

## Figures and Tables

**Table 1:** IC_50_ value of samples on HCT116 and CCD112.

Samples	IC_50_ (mg/mL)	
	HCT116	CCD112
PCA	0.148	0.224
MMT/CR/Fe_3_O_4_-NCs	1.500	1.630
MMT/CR/Fe_3_O_4_-PCA	0.734	0.841

Abbreviations: IC_50_ = inhibitory concentration that kills 50% of the tested cell line.

## Abbreviations

MMT	Montmorillonite
CR	Carrageenan
NCs	Nanocomposites
NPs	Nanoparticles
PCA	Protocatechuic acid
